# Electrochemical oxidation of cholesterol

**DOI:** 10.3762/bjoc.11.45

**Published:** 2015-03-25

**Authors:** Jacek W Morzycki, Andrzej Sobkowiak

**Affiliations:** 1 Institute of Chemistry, University of Białystok, ul. Ciołkowskiego 1K, 15-245 Białystok, Poland; 2 Faculty of Chemistry, Rzeszów University of Technology, P.O. Box 85, 35-959 Rzeszów, Poland

**Keywords:** allylic oxidation, cholesterol, electrochemical halogenation, electrochemical oxidation

## Abstract

Indirect cholesterol electrochemical oxidation in the presence of various mediators leads to electrophilic addition to the double bond, oxidation at the allylic position, oxidation of the hydroxy group, or functionalization of the side chain. Recent studies have proven that direct electrochemical oxidation of cholesterol is also possible and affords different products depending on the reaction conditions.

## Introduction

Cholesterol is the most common steroid in the mammalian body. It is necessary to ensure a proper membrane permeability and fluidity. It also serves as a precursor for the biosynthesis of steroid hormones, bile acids, and vitamin D [1]. The chemical oxidation of cholesterol is a crucial reaction in the synthesis of many compounds that are of pharmaceutical importance [2–3]. At the close of the previous century, special attention started being paid to cholesterol oxidation products [4]. Since then, these compounds have constantly drawn the attention of biochemists and medicinal chemists. Cholesterol that is present in food of animal origin undergoes autoxidation during processing as well as during storage, thus it yields toxic products. These are formed due to oxidation reactions caused by the contact with oxygen, the exposure to sunlight, heating treatments, etc. [5–7]. Furthermore, they can be generated in the human organism through different oxidation processes, some of which require enzymes. Cholesterol oxidation products cause many diseases, coronary heart disease and atherosclerosis being among the most common in modern societies [8–11]. Cholesterol oxidation products have also been proven to exhibit cytotoxicity as well as apoptotic and pro-inflammatory effects [12–13]. Therefore, in-depth studies on cholesterol oxidation may allow for significant advances in cholesterol biology and chemistry.

## Review

### General remarks

A series of physiological actions in both humans and animals are caused by chemical oxidation, photooxidation or enzymatic oxidation of cholesterol. Despite the importance of these processes, cholesterol has been regarded as an electrochemically inactive compound [14]. However, during the last two decades the electrochemical oxidation of cholesterol, direct and indirect, was studied intensively. The first reports on cholesterol oxidation concerned indirect electrochemical methods with redox agents as electron mediators [15–16]. In these methods, cholesterol (Chol) forms adducts with the oxidized redox agents and then reacts, affording the products (P). The reduced forms of redox agents also produced in these processes are electrochemically regenerated. The indirect electrochemical reaction can be outlined as follows [17]:

M_red_ → M_ox_ + ne^−^,

M_ox_ + Chol → M_ox_·Chol,

M_ox_·Chol → M_red_ + P,

where M_red_ and M_ox_ are the redox agents in the reduced and oxidized states, respectively.

**Figure 1:**
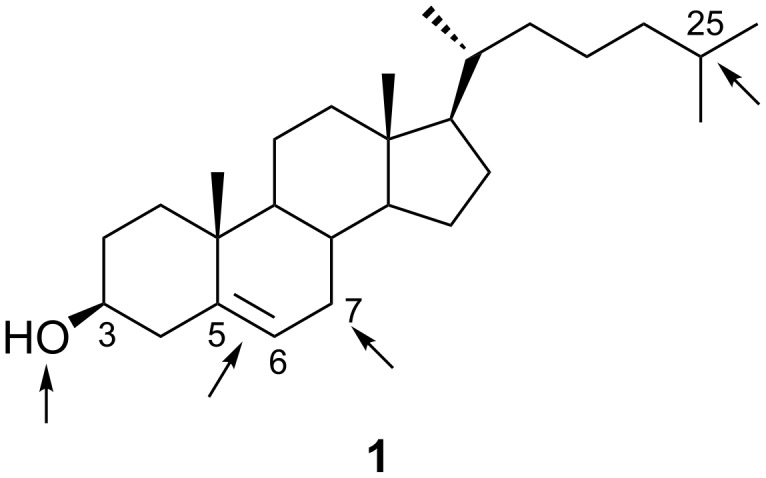
Preferential sites of cholesterol electrooxidation.

From a chemical point of view, cholesterol (**1**) is a homoallylic alcohol with a relatively large hydrophobic part. The preferential sites of cholesterol electrooxidation are shown in Figure 1. These are the hydroxy group at C3, the C5–C6 double bond, the allylic positions (particularly C7), and the tertiary positions (mainly in the side chain at C25). The multiple potential sites of chemical or electrochemical oxidation of cholesterol lower the reaction yields. The search for highly regio- and stereoselective reactions is a challenging problem. The yields of reactions are also low due to various consecutive reactions. To avoid this problem chemists frequently stop the reactions before completion. In many cases, the yields given in this article are not compensated for a low conversion.

All compounds throughout this review are assigned to Arabic numerals. Sometimes, the only difference between two compounds is the presence of an acetyl group at C3. In that case, the two compounds have the same numeral, but the acetylated derivative is amended by the letter “a”, e.g., cholesterol (**1**) and cholesteryl acetate (**1a**).

### Indirect electrochemical oxidation of cholesterol

**Scheme 1:**
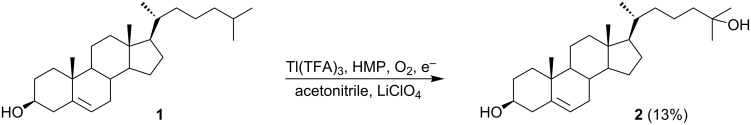
Functionalization of the cholesterol side chain.

The selective oxidation of saturated hydrocarbons by dioxygen and hydrogen peroxide remains a challenging problem in chemistry and biology [18–21]. In analogy of the oxidation of cholesterol in the human body the Takayama group [22] has employed the process of dioxygen activation by Tl(II), which was electrochemically generated by the cathodic reduction of the Tl(III) hematoporphyrin (HMP) complex. The system produced activated oxygen species that regioselectively oxidized the tertiary C–H bonds. The reactions were carried out under constant current conditions in aqueous acetonitrile with Tl(TFA)_3_, HMP, LiClO_4_ and by bubbling with O_2_ gas with platinum electrodes in an undivided cell. The starting cholesterol was transformed to 25-hydroxycholesterol (**2**) in 13% yield (Scheme 1). The statement that the Tl(II)/HMP/O_2_ adduct, suggested to be a radical in nature, is responsible for cholesterol oxidation was based on the following observations. The electrolysis performed in the divided cell indicated that the oxidation of cholesterol took place in the cathodic compartment. The oxidation did not occur without the electrochemical reduction of Tl(III), which suggests that Tl(III) cannot activate dioxygen. In addition, the replacement of dioxygen by hydrogen peroxide gave a mixture of oxidation products. This indicates that dioxygen was not electrochemically reduced to hydrogen peroxide, which could act as an oxidant. It was also observed that the replacement of Tl(III) with Fe(III) caused a decrease in the reaction yield and the replacement of HMP with tetraphenylporphyrin or its derivatives resulted in product mixtures.

**Scheme 2:**
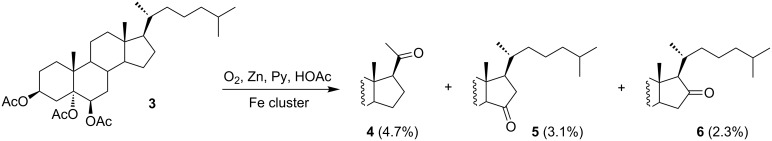
Oxidation of cholestane-3β,5α,6β-triol triacetate (**3**) with the Gif system.

Many investigations on oxygenation reactions have been carried out by using a simple, readily available reagent system mimicking monooxygenase enzymes. An interesting system for the oxidation of aliphatic hydrocarbons, consisting of oxygen, powdered zinc, pyridine, acetic acid, and a catalytic amount of an iron cluster, was described in the 1980s by Barton et al. [23]. The system (Gif system) showed an unusual regioselectivity – the secondary positions were preferentially oxidized. An identical selectivity was also observed in an electrochemical version of the reaction (Gif–Orsay system), in which the reducing agent for oxygen (zinc) was replaced with electroreduction [24–25]. The chemical system was also applied to the oxidation of a side chain (Scheme 2) in protected cholesterol **3** [26], affording the corresponding 20-oxo derivative **4** (4.7%; yields are not corrected for recovered starting material) as the main product in addition to other products (e.g. 15-oxo **5**, 16-oxo **6** and other oxo-steroids). The electrooxidation of cholesterol derivatives also produced ketones but the detailed product analysis was not reported. It seems that the active species for the ketone formation in Gif systems is Fe(V)=O formed by heterolytic O–O-bond cleavage in Fe(III)–O–OH. There is no direct evidence for the existence of such species in Gif-systems and it was not spectroscopically characterized. However, the arguments for the oxoiron(V) species as an active oxidant has been recently summarized by Que [27]. In Gif systems pyridine is not only used as a solvent but it also acts as a ligand on the iron complex [28].

**Scheme 3:**
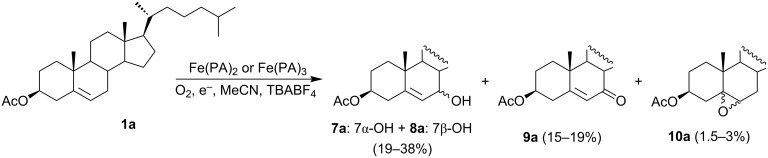
Electrochemical oxidation of cholesteryl acetate (**1a**) with dioxygen and iron–picolinate complexes.

Different Fe(II) or Fe(III) picolinate (PA) and dipicolinate (DPA) complexes as catalysts of oxygenation reactions have been studied by the Sawyer [29–31], Barton [31–32], and Kotani–Takeya [33–34] groups. The oxidation of cholesteryl acetate with hydrogen peroxide or peracetic acid catalyzed by these complexes varied depending on the solvent used and the reaction conditions. To avoid the use of hazardous peroxy reagents, an electrochemical system was invented, which can be considered as analogical to the Gif-system. In this approach, hydrogen peroxide was exchanged for a process of dioxygen activation with electrochemically generated [Fe^II^(PA)_3_OH_2_]^−^ ions. With the system, stereoselective allylic hydroxylation of cholesteryl acetate was carried out [35]. The H-shaped one-compartment glass cell equipped with platinum mesh electrodes (cathode and anode) was filled with a cholesteryl acetate (**1a**) solution in acetonitrile containing ammonium tetrafluoroborate as a supporting electrolyte. The constant potential technique mainly afforded 7-hydroxylated products (**7a** and **8a**; 19–38%), along with the 7-oxo product **9a** (15–19%). In addition to these products, epoxide **10a** (1.5–3%) was also formed under constant current conditions (Scheme 3). Irrespective of the iron complex used, the reactions afforded the 7α-hydroxylated product **7a** in a large excess. The authors postulated the formation of a dimeric Fe(III)–Fe(V) manifold complex, (H_2_O)(PA)_2_Fe(III)–O–Fe(V)=O(PA)_2_, as an active intermediate that would work as a monooxygenating species for cholesteryl acetate.

Indirect electrochemical oxidation of cholesterol was also reported by Wu et al. [36]. The electrolysis was performed under galvanostatic conditions at a platinum electrode in DMF containing 6% water with NaBr as a supporting electrolyte. A mixture of products was formed, among them “3,5,6-trihydroxycholesterol” (probably cholesta-3β,5ξ,6ξ-triol), 7-oxocholesterol (**9**), 5,6-epoxycholesterol (**10**), and 7-ketocholesterol were identified. However, the yields of the products were not reported. Based on cyclic voltammetry and rotating ring-disk electrode measurements the authors suggested that Br^−^ is electrochemically oxidized through Br to BrO^−^ and that the latter is the oxidizing agent for cholesterol.

The oxidation of cholesterol by electrochemically generated superoxide radical anion (O_2_^·−^) in acetonitrile was described in 1997 [37]. It was established that in dry solution, with water content below 4%, no products were observed. However, cholesterol was oxidized to 7-oxocholesterol (**9**), 7α-hydroxycholesterol (**7**), and 7β-hydroxycholesterol (**8**) when hydrogen peroxide was combined with superoxide solution in water matrix. The authors suggested that under these conditions the rate of the disproportionation reaction of superoxide radical anion is increased, and the formation of more reactive oxygen species such as hydroxyl radical or singlet oxygen is possible.

**Scheme 4:**
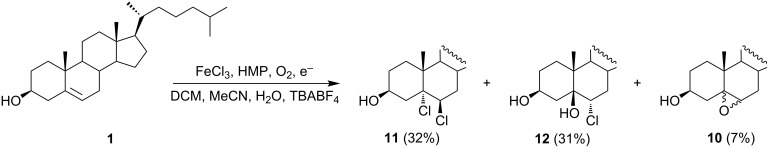
Electrochemical chlorination of cholesterol catalyzed by FeCl_3_.

A stereoselective electrochemical method of the chlorination of steroidal Δ^5^-olefins was described by Takayama et al. [38]. The oxidation of cholesterol was carried out in an undivided cell under constant current conditions in CH_2_Cl_2_/MeCN/H_2_O (2:2:1), the cathode and anode being platinum plates. The electrolysis was performed in the presence of FeCl_3_ and hematoporphyrin (HMP), and dioxygen was constantly delivered to the cell. Three products, 5α,6β-dichlorocholestan-3β-ol (**11**), 6α-chlorocholestane-3β,5ß-diol (**12**), and epoxide (**10**), as a 1:3 mixture of α and β-isomers, were obtained in 32, 31, and 7% yields, respectively (Scheme 4). It is noteworthy that the reaction afforded a single stereoisomer of **11** or **12**.

Several important observations provided in the paper can give an idea on a plausible mechanism of cholesterol oxidation. First, in the absence of HMP and O_2_ only compounds **11** (20%) and **12** (43%) were formed. Second, during the reaction with hydrogen peroxide as an oxidant under non-electrochemical conditions no products were detected, which indicates that the role of dioxygen is not the source of electrochemically generated H_2_O_2_. Furthermore, the epoxide **10** was not converted into chlorohydrin **12** under the reaction conditions, which implies it was not formed through an epoxide intermediate. Finally, no products were formed when the electrolysis was performed in a divided cell. It seems that a chlorine species (e.g. Cl^δ+…^FeCl_4_^δ−^), produced from anodically generated chlorine (dichloromethane was the likely source), initiated the sequence of reactions leading to cholesterol oxidation products.

A very similar result was obtained by Kowalski et al. [39]. They carried out the electrolysis of cholesterol in dichloromethane by using a divided cell, the cathode of which was placed in a glass tube with a glass frit. No metal chlorides were added, but again the major products were dichlorides, 5α,6β-dichlorocholestan-3β-ol (**11**, 14%) and its 5β,6α-isomer (9%), as well as chlorohydrin **12** (6%) and 3β-chlorocholest-5-ene (**1c**, 4%). It is clear that dichloromethane was the chlorine source since no additives were employed. The observed results may be explained by assuming a cathodic reduction of dichloromethane to chloride ions, followed by their diffusion to the anodic compartment, and electrooxidation to chlorine which reacted with cholesterol.

**Scheme 5:**
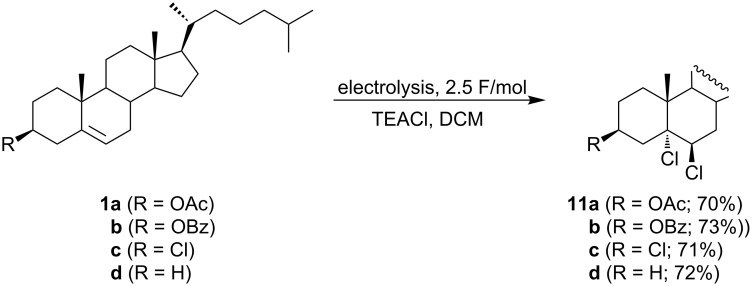
Electrochemical chlorination of Δ^5^-steroids.

An efficient electrochemical chlorination of some Δ^5^-steroids (Scheme 5) was reported by Milisavljević and Vukićević [40]. The electrolyses were carried out in dichloromethane under galvanostatic conditions with a graphite anode in an undivided cell. The supporting electrolyte tetraethylamonium chloride was a source of chloride ions, which were anodically oxidized to chlorine. The electrophilic addition of chlorine to the double bond of the investigated compounds cholesteryl acetate (**1a**), cholesteryl benzoate (**1b**), 3β-chloro-5-cholestene (**1c**), and 5-cholestene (**1d**), gave the corresponding 5α,6β-dichloro steroids in good yields (70–73%). However, cholesterol produced a complex mixture of products under the same reaction conditions.

**Scheme 6:**
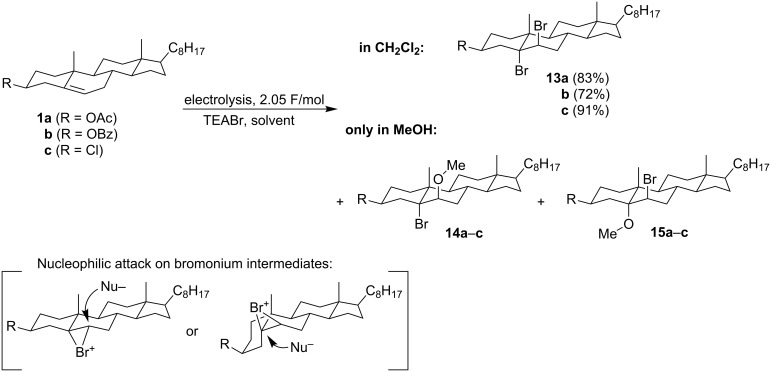
Electrochemical bromination of Δ^5^-steroids in different solvents.

The same authors also studied the electrochemical bromination of similar cholesterol derivatives **1a**–**c** (Scheme 6) [41]. The electrolyses were performed in a divided cell with a platinum anode in different solvents under constant current conditions. The supporting electrolyte tetraethylammonium bromide was used as a source of bromide ions, which were oxidized to give bromine. In aprotic solvents (dichloromethane, acetonitrile or acetic anhydride) only the corresponding 5α,6β-dibromocholestanes **13a**–**c** were formed in high yields. In methanol, however, in addition to dibromides **13a**–**c**, the corresponding regioisomeric pairs of 5α-bromo-6β-methoxy- and 5α-methoxy-6β-bromocholestanes (**14a**–**c** and **15a**–**c**) in a ratio of 3:1 were produced. The reactions apparently proceeded via a tricentric bromonium ion. Since the subsequent nucleophilic attack on this intermediate must occur from the back, the overall stereochemistry of the addition must be *anti*. It seems that in the first step an electrophilic attack of Br^+^ from the α side prevails. The diaxial opening of the bromonium intermediate with bromide yields the same dibromo product **13a**–**c**, irrespective which bromonium ion (α or β) is actually formed. When this intermediate is attacked by methanol, the product structure **14a**–**c** or **15a**–**c** depends on the configuration of the bromonium ion.

### Direct electrochemical oxidation of cholesterol

**Scheme 7:**
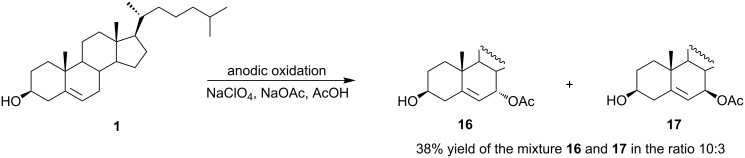
Direct electrochemical acetoxylation of cholesterol at the allylic position.

The first direct electrochemical oxidation of cholesterol was reported only in 2005 [42]. The preparative electrolysis was performed in glacial acetic acid on a platinum anode under constant current control in a divided cell. The reaction afforded two major products, 7α-acetoxycholesterol (**16**) and 7β-acetoxycholesterol (**17**), in a ratio of 10:3 (Scheme 7).

However, several byproducts were also formed. Voltammetric measurements indicated that the cholesterol oxidation process is controlled by the rate of the electron transfer. It was proven that the oxidation occurs at the allylic position. The C7 carbocation is formed by a two-electron transfer and then a nucleophile (acetate) is added to this intermediate, preferentially from the less sterically hindered α-side.

**Scheme 8:**
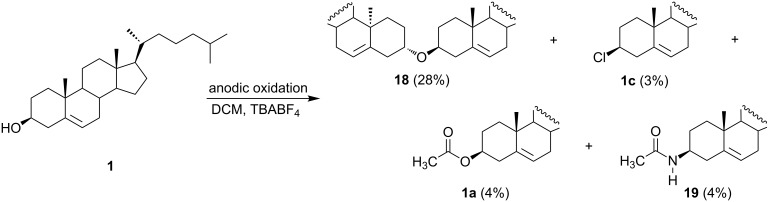
Direct anodic oxidation of cholesterol in dichloromethane.

An interesting product of the electrochemical oxidation of cholesterol [39], i.e. dicholesteryl ether **18**, was obtained in 28% yield during the electrolysis carried out in a divided cell in dichloromethane as a solvent (Scheme 8). Except for the major product, the formation of small amounts of cholesteryl acetate (**1a**, 4%), cholesteryl chloride (**1c**, 3%), and *N*-acetylcholesterylamine (**19**, 4%) were also observed. The formation of cholesteryl chloride (**1c**) was explained by the possibility of cathodic dichloromethane reduction yielding chloride ions, which can migrate to the anodic compartment. To prevent the cathodic reduction of dichloromethane a small amount of glacial acetic acid was added to the cathodic compartment, but its leakage to the anodic part of the cell was responsible for the appearance of cholesteryl acetate (**1a**) among the oxidation products. To avoid this problem the anodic and cathodic parts of the cell were connected with an electrolytic bridge, which contained acetonitrile among other components to increase conductivity. However, its diffusion to the anode can be attributed to the formation of *N*-acetylcholesterylamine (**19**).

**Scheme 9:**
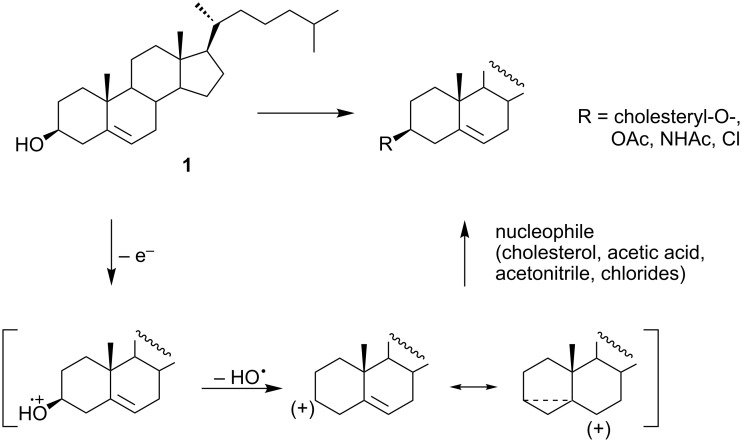
A plausible mechanism of the electrochemical oxidation of cholesterol in dichloromethane.

All observed products can be formed through a common intermediate. It seems that the first step of the reaction is a one-electron transfer from the oxygen atom of cholesterol to the anode (Scheme 9). The heterolytic cleavage of the C3–O bond in the resulting radical cation leads to the formation of a hydroxyl radical and the steroidal carbocation. Such a mesomerically stabilized homoallylic carbocation can react with any nucleophile present in the reaction mixture. In the absence of better nucleophiles it reacts with cholesterol to give dicholesteryl ether **18**.

**Scheme 10:**
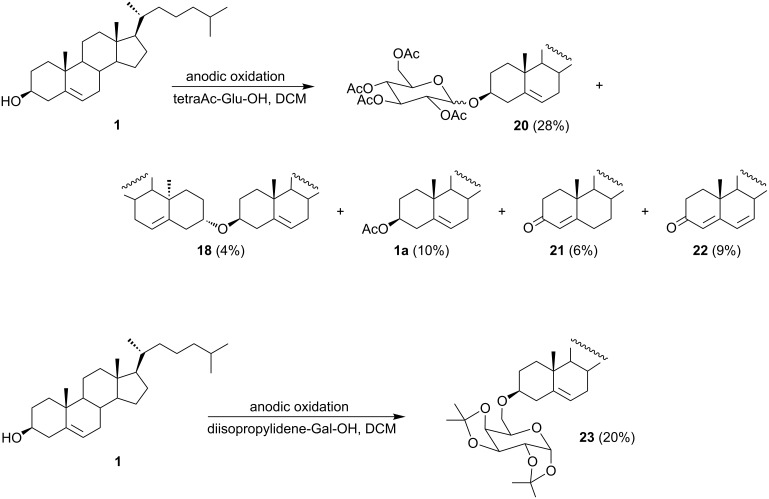
The electrochemical formation of glycosides and glycoconjugates.

Later, the electrochemical system was improved by replacing the functionality of the bridge with a divided H-cell with anionite (Dowex) placed in the cathodic compartment to bind chloride anions which are formed by the reduction of dichloromethane. The formation of byproducts was diminished with this system. The presented system proved suitable for the electrochemical glycosylation of 3β-hydroxy-Δ^5^-steroids [43]. In this case, 2,3,4,6-tetra-*O*-acetyl-D-glucopyranose was used as a nucleophile (Scheme 10). The anodic oxidation of cholesterol (**1**) carried out in dichloromethane in the presence of the sugar used in excess afforded glycoside **20** (28%) as a 1:1 mixture of α and β-anomers, accompanied by a number of by-products such as cholesteryl acetate (**1a**, 10%), dicholesteryl ether (**18**, 4%), cholest-4-en-3-one (**21**, 6%), and cholesta-4,6-dien-3-one (**22**, 9%).

An analogous reaction carried out with 1,2:3,4-di-*O*-diisopropylidene-α-D-galactopyranose yielded glycoconjugate **23** with an ether bond between the sugar and steroid molecules. In further studies on the preparation of glycoconjugates from 3β-hydroxy-Δ^5^-steroids, various derivatives were applied, such as thioethers [44], diphenylphosphates [45], trichloroacetimidates [45], and i-steroids [46].

**Scheme 11:**
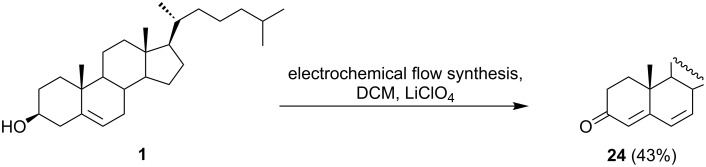
Efficient electrochemical oxidation of cholesterol to cholesta-4,6-dien-3-one (**24**).

Cholesterol has been shown to be electrochemically oxidized (Scheme 11) in acetonitrile containing LiClO_4_ at a carbon electrode [47] to give cholesta-4,6-dien-3-one (**24**). The electrolysis conditions were optimized on a laboratory synthetic scale [48]. The use of a flow cell equipped with a carbon fiber working electrode, allowed for the efficient production (43% yield) of cholesta-4,6-dien-3-one (**24**) during electrolysis under potentiostatic conditions at 1.9 V vs Ag/AgCl electrode. This result is rather surprising since cholesta-4,6-dien-3-one was not previously reported as a major product during electrochemical oxidation of cholesterol. The product was formed through a four-electron, four-proton electrochemical process, but no explanation was given for the selectivity observed.

### Enzymatic and non-enzymatic methods of cholesterol determination with electrochemical detection

The development of fast and reliable methods of cholesterol determination in the human body and in processed food is still a demanding problem. Since this review article is mainly focused on the electrochemical transformations (direct or indirect) of cholesterol, the analytical aspects of cholesterol oxidation are only briefly mentioned. The cholesterol reactions that lie behind the analytical procedures are frequently unknown or are largely neglected by their authors. To date, many analytical methods have been developed to quantitate cholesterol contents, including spectrophotometry and HPLC, sometimes with electrochemical detection [49].

The majority of cholesterol determination methods however, take advantage of electrochemical biosensors based on cholesterol oxidase immobilized on an electrode surface. Cholesterol oxidase is an enzyme that catalyzes the reaction between cholesterol and dioxygen to produce cholest-4-en-3-one and hydrogen peroxide [50]. Then, the cholesterol level can be determined from an amperometric response, which can either be measured as a decrease in the dioxygen electroreduction current or, more frequently, from the hydrogen peroxide reduction or oxidation current. However, cholesterol in blood is mainly present in form of its fatty acid esters. If the total cholesterol amount is needed, the cholesterol esters must be hydrolyzed prior to analysis by the use of cholesterol esterase [51]. A poor stability of the enzymes and an influence of various factors (e.g., temperature and pH) on their performance limit a practical application of these methods. Therefore, the procedure of an enzyme immobilization on electrode surface is a crucial step for biosensors stability and efficiency.

### Enzymatic oxidation of cholesterol with electrochemical detection

There are numerous reports on these methods and their detailed discussion is beyond the scope of the present review. Therefore, we limited ourselves to these publications, which include a comparison of the applied methods and have been published only recently. Up to date the most popular method is the immobilization of an enzyme on the electrode covered with different conducting polymers often embedded with carbon nanotubes and/or metal nanoparticles [52–56]. Chitosan, a naturally occurring biopolymer, has also been utilized for sensor fabrication [57–58]. Cholesterol oxidase has been immobilized on carbon nanotubes [59], metal nanoparticles [60] or graphene [61], and additionally decorated with metal nanoparticles [62] or modified with ionic liquids [63]. The application of composite electrodes, including silica sol–gel matrix with Prussian Blue [64], carbon nanotubes with zinc oxide nanoparticles [65], and zinc oxide nanorods directly grown on silver [66], has also been reported on. The “cholesterol self-powered biosensor” [67], in which the cathodic process is determined by cholesterol oxidase and on an anode phenothiazine-mediated oxidation of cholesterol as well as immobilization of cholesterol oxidase and cholesterol esterase onto thulium oxide [68] have been found as an alternative for biosensors which operate in blood.

### Non-enzymatic indirect cholesterol detection with electrochemical techniques

Non-enzymatic approaches toward cholesterol detection exploiting an electrochemical route of sensing, which have a distinct advantage over conventional enzymatic processes, have recently been developed. Some of these methods are based on the indirect electrochemical oxidation of cholesterol by using bromine species in organic media [69–70]. In a recent method, methylene blue formed an inclusion complex with β-cyclodextrin functionalized graphene and emerged as a cholesterol sensing matrix. Methylene blue was then replaced by the cholesterol molecule and moved out in the buffer solution, where it was detected electrochemically by using the differential pulse voltammetric technique [71]. An interesting technique for non-enzymatic sensors represents molecularly imprinted self-assembled monolayers. In this approach, the layer containing organic compounds together with cholesterol is deposited on an electrode surface. Then, the cholesterol is removed from the layer, and the oxidation current of ferrocyanide is measured. Next, the electrode is placed into a solution containing cholesterol, which is adsorbed in the empty spots and the oxidation current of ferrocyanide is measured again. The difference in the current values is proportional to the concentration of cholesterol [72–74]. These sensors have been proved to be useful for analyzing food samples [74].

### Non-enzymatic direct electrochemical oxidation of cholesterol

The third class represents sensors based on the direct electrochemical oxidation of cholesterol. The authors claim that the process occurs on nanoporous electrodes such as Pt [75], Ag [76], Au/Pt [77], and Cu_2_S [78]. However, apart from the increase of the registered current, there is no evidence that cholesterol is electrochemically oxidized. The assumption that cholesterol is directly electrooxidized seems surprising, as the observed process occurs at relatively negative potentials (0 to +0.4 V vs SCE). Definitely, the mechanism of the process needs to be established.

There is a worldwide effort toward the development of bioanalytical devices which can be used for the detection, the quantification and the monitoring of specific chemical species. The design of mixed biocatalyst pathways for the comprehensive oxidation of cholesterol, and, at the same time, the acquisition of frequent answers with an individual application for biosensorics is of major interest. Sophisticated sensing arrangements including single and complex selective agents may be expected to contribute to clinical chemistry.

## Conclusion

Cholesterol has been regarded as an electrochemically inactive compound for a long time. Since the 1990s several methods have been developed for the indirect oxidation of cholesterol. The active species in these methods were different reactive oxygen species, various hypervalent transition metal–oxo and metal–peroxo species, dihalogens, hypohalites, and enzymes. The first direct electrochemical oxidation was reported only in 2005. The electrochemical oxidation of cholesterol may occur at the C3-hydroxy group, at the C5–C6 double bond, at the allylic position, and at the side chain (particularly at the tertiary position C25). Interestingly, the particular progress of the reaction depends on the reaction conditions, including the solvent, the supporting electrolyte, the mediator, the electrode material, and the potential applied. The yields of cholesterol oxidation products are rather low, often less than 30%. This may be caused by the high oxidation potential of cholesterol and the necessity to operate at a relatively high positive potential. Moreover, due to the hydrophobic properties of cholesterol it is necessary to use non-polar solvents, e.g., dichloromethane, which lowers the conductivity of the supporting electrolyte. Therefore, the galvanostatic regime of electrolyzes is often applied, which favors the occurrence of side reactions. The cholesterol oxidation products are often adsorbed at the electrode surface, which lowers the effectiveness of the electrochemical process. However, in some cases, reasonable yields of products are obtained and these reaction conditions may be of interest in practice.

## References

[1] Morzycki J W (2014). Steroids.

[2] Djerassi C (1963). Steroid Reactions: An Outline for Organic Chemists.

[3] Fried J, Edwards J A (1972). Organic Reactions in Steroid Chemistry.

[4] Schroepfer G J (2000). Physiol Rev.

[5] Wentworth P, Nieva J, Takeuchi C, Galve R, Wentworth A D, Dilley R B, DeLaria G A, Saven A, Babior B M, Janda K D (2003). Science.

[6] Brinkhorst J, Nara S J, Pratt D A (2008). J Am Chem Soc.

[7] Derewiaka D, Molińska E (2015). Food Chem.

[8] Umetani M, Ghosh P, Ishikawa T, Umetani J, Ahmed M, Mineo C, Shaul P W (2014). Cell Metab.

[9] Lim W L F, Martins I J, Martins R N (2014). J Genet Genomics.

[10] Björkhem I (2013). Biochimie.

[11] Sottero B, Gamba P, Gargiulo S, Leonarduzzi G, Poli G (2009). Curr Med Chem.

[12] De Weille J, Fabre C, Bakalara N (2013). Biochem Pharmacol.

[13] Miyoshi N, Iuliano L, Tomono S, Ohshima H (2014). Biochem Biophys Res Commun.

[14] Görög S (1983). Quantitative Analysis of Steroids.

[15] Steckhan E (1986). Angew Chem, Int Ed.

[16] Shono T, Matsumura Y, Inoue K (1984). J Am Chem Soc.

[17] Simonet J, Pilard J F, Lund H, Hammerich O (2001). Electrogenerated reagents. Organic Electrochemistry.

[18] Barton D H R, Doller D (1992). Acc Chem Res.

[19] Bovicelli P, Lupattelli P, Mincione E, Prencipe T, Curci R (1992). J Org Chem.

[20] Sawyer D T, Sobkowiak A, Matsushita T (1996). Acc Chem Res.

[21] Barton D R H (1998). Tetrahedron.

[22] Maki S, Konno K, Takayama H (1997). Tetrahedron Lett.

[23] Barton D H R, Boivin J, Gastiger M, Morzycki J W, Hay-Motherwell R S, Motherwell W B, Ozbalik N, Schwartzentruber K M (1986). J Chem Soc, Perkin Trans 1.

[24] Balavoine G, Barton D H R, Boivin J, Gref A, Le Coupanec P, Ozbalik N, Pestana J A X, Rivière H (1988). Tetrahedron.

[25] Barton D H R, Sobkowiak A (1996). New J Chem.

[26] Barton D H R, Gökturk A K, Morzycki J W, Motherwell W B (1985). J Chem Soc, Perkin Trans 1.

[27] Oloo W N, Feng Y, Iyer S, Parmelee S, Xue G, Que L (2013). New J Chem.

[28] Barton D H R, Hu B, Li T, MacKinnon J (1996). Tetrahedron Lett.

[29] Sheu C, Richert S A, Cofre P, Ross B, Sobkowiak A, Sawyer D T, Kanofsky J R (1990). J Am Chem Soc.

[30] Kang C, Sobkowiak A, Sawyer D T (1994). Inorg Chem.

[31] Sheu C, Sobkowiak A, Zhang L, Ozbalik N, Barton D H R, Sawyer D T (1989). J Am Chem Soc.

[32] Barton D H R, Salgueiro M C, MacKinnon J (1997). Tetrahedron.

[33] Takeya T, Egawa H, Inoue N, Miyamoto A, Chuma T, Kotani E (1999). Chem Pharm Bull.

[34] Okamoto I, Funaki W, Nobuchika S, Sawamura M, Kotani E, Takeya T (2005). Chem Pharm Bull.

[35] Okamoto I, Funaki W, Nakaya K, Kotani E, Takeya T (2004). Chem Pharm Bull.

[36] Wu X, Song C, Cheng F, Zhang W (1992). J Electroanal Chem.

[37] Lee J H, Shoeman D W, Kim S-S, Csallany A S (1997). Int J Food Sci Nutr.

[38] Maki S, Konno K, Ohba S, Takayama H (1998). Tetrahedron Lett.

[39] Kowalski J, Łotowski Z, Morzycki J W, Płoszyńska J, Sobkowiak A, Wilczewska A Z (2008). Steroids.

[40] Milisavljević S, Vukićević R D (2004). J Serb Chem Soc.

[41] Milisavljević S, Wurst K, Laus G, Vukićević M D, Vukićević R D (2005). Steroids.

[42] Kowalski J, Płoszyńska J, Sobkowiak A, Morzycki J W, Wilczewska A Z (2005). J Electroanal Chem.

[43] Morzycki J W, Łotowski Z, Siergiejczyk L, Wałejko P, Witkowski S, Kowalski J, Płoszyńska J, Sobkowiak A (2010). Carbohydr Res.

[44] Tomkiel A M, Brzezinski K, Łotowski Z, Siergiejczyk L, Wałejko P, Witkowski S, Kowalski J, Płoszyńska J, Sobkowiak A, Morzycki J W (2013). Tetrahedron.

[45] Tomkiel A M, Kowalski J, Płoszyńska J, Siergiejczyk L, Łotowski Z, Sobkowiak A, Morzycki J W (2014). Steroids.

[46] Tomkiel A M, Biedrzycki A, Płoszyńska J, Naróg D, Sobkowiak A, Morzycki J W (2015). Beilstein J Org Chem.

[47] Hosokawa Y-Y, Hakamata H, Murakami T, Aoyagi S, Kuroda M, Mimaki Y, Ito A, Morosawa S, Kusu F (2009). Electrochim Acta.

[48] Hosokawa Y-Y, Hakamata H, Murakami T, Kusu F (2010). Tetrahedron Lett.

[49] Hojo K, Hakamata H, Ito A, Kotani A, Furukawa C, Hosokawa Y-Y, Kusu F (2007). J Chromatogr, A.

[50] Pollegioni L, Piubelli L, Molla G (2009). FEBS J.

[51] Stępień A E, Gonchar M (2013). Acta Biochim Pol.

[52] Rahman M M, Li X, Kim J, Lim B O, Ahammad A J S, Lee J-J (2014). Sens Actuators, B.

[53] Fang K-C, Chu C-H, Hsu C-P, Kang Y-W, Fang J-Y, Hsu C-H, Huang Y-F, Chen C-C, Li S-S, Yeh J A (2014). Appl Phys Lett.

[54] Cai X, Gao X, Wang L, Wu Q, Lin X (2013). Sens Actuators, B.

[55] Kakhki S, Barsan M M, Shams E, Brett C M A (2013). Anal Methods.

[56] Prakash S, Chakrabarty T, Singh A K, Shahi V K (2013). Biosens Bioelectron.

[57] Srivastava M, Srivastava S K, Nirala N R, Prakash R (2014). Anal Methods.

[58] Charan C, Shahi V K (2014). J Appl Electrochem.

[59] Tong Y, Li H, Guan H, Zhao J, Majeed S, Anjum S, Liang F, Xu G (2013). Biosens Bioelectron.

[60] Ahmadalinezhad A, Chen A (2011). Biosens Bioelectron.

[61] Manjunatha R, Suresh G S, Melo J S, D’Souza S F, Venkatesha T V (2012). Talanta.

[62] Cao S, Zhang L, Chai Y, Yuan R (2013). Talanta.

[63] Gholivand M B, Khodadadian M (2014). Biosens Bioelectron.

[64] Li J, Peng T, Peng Y (2003). Electroanalysis.

[65] Gupta V K, Norouzi P, Ganjali H, Faridbod F, Ganjali M R (2013). Electrochim Acta.

[66] Israr M Q, Sadaf J R, Asif M H, Nur O, Willander M, Danielsson B (2010). Thin Solid Films.

[67] Sekretaryova A N, Beni V, Eriksson M, Karyakin A A, Turner A P F, Vagin M Y (2014). Anal Chem.

[68] Singh J, Roychoudhury S, Srivastava M, Solanki P R, Lee D W, Lee S H, Malhotra B D (2014). Nanoscale.

[69] Tsierkezos N G, Ritter U (2014). Phys Chem Liq.

[70] Chiang W-H, Chen P-Y, Nien P-C, Ho K-C (2011). Steroids.

[71] Agnihotri N, Chowdhury A D, De A (2015). Biosens Bioelectron.

[72] Piletsky S A, Piletskaya E V, Sergeyeva T A, Panasyuk T L, El’skaya A V (1999). Sens Actuators, B.

[73] Shiigi H, Matsumoto H, Ota I, Nagaoka T (2008). J Flow Injection Anal.

[74] Nagaoka T, Tokonami S, Shiigi H, Matsumoto H, Takagi Y, Takahashi Y (2012). Anal Sci.

[75] Lee Y-J, Kim J-D, Park J-Y (2009). J Korean Phys Soc.

[76] Li Y, Bai H, Liu Q, Bao J, Han M, Dai Z (2010). Biosens Bioelectron.

[77] Lee Y-J, Park J-Y (2010). Biosens Bioelectron.

[78] Ji R, Wang L, Wang G, Zhang X (2014). Electrochim Acta.

